# The Impact of Social Support on Dementia Caregivers’ Psychological Well-being: Is Online Comparable to Offline?

**DOI:** 10.1093/geroni/igab046.2972

**Published:** 2021-12-17

**Authors:** Eun-Hye Yi

**Affiliations:** Indiana State University, United States

## Abstract

Along with the groundbreaking development of communication technology, caregivers have migrated to online platforms to seek help. Social support theory, including main effect and moderation effect models, has provided a framework to understand the association among caregivers’ stress, support from others, and their well-being. Despite the prevalence of online use for seeking help among caregivers, studies on the use of online social support within the context of caregivers’ stressors and well-being are still underdeveloped. Guided by social support theory, this study aimed to examine the association of online social support (OnSS) and caregivers’ mental health (MH) as compared with offline social support (OffSS). A subsample of caregivers of persons with Alzheimer’s from the Health Information National Trends Survey from 2017-2018 was selected (n=264). For an analysis, ordered logistic regression models with Jackknife estimation methods were applied using Stata 15.1SE. First, OffSS had a positive direct association with caregivers’ MH (Odds Ratio=12.48, p<.05) while OnSS did not. Next, the moderation effect model analysis found that OffSS interacted with caregiving burden while OnSS interacted with life stressors. The MH of caregivers who are in less favorable situations, such as working part-time while caring for a person with Alzheimer’s, living with economic hardship, and having health problems, tended to be significantly affected by OnSS. Identifying the different roles of OnSS and OffSS for caregivers’ MH, the findings of this study call for more attention to developing novel strategies and sensitive approaches to support family caregivers, especially those who fall in underserved groups.

